# Development of Nanogel Loaded with Lidocaine for Wound-Healing: Illustration of Improved Drug Deposition and Skin Safety Analysis

**DOI:** 10.3390/gels8080466

**Published:** 2022-07-26

**Authors:** Amena Ali, Abuzer Ali, Mohammad Akhlaquer Rahman, Musarrat Husain Warsi, Mohammad Yusuf, Prawez Alam

**Affiliations:** 1 Department of Pharmaceutical Chemistry, College of Pharmacy, Taif University, P.O. Box 11099, Taif 21944, Saudi Arabia; 2 Department of Pharmacognosy, College of Pharmacy, Taif University, P.O. Box 11099, Taif 21944, Saudi Arabia; abuali@tu.edu.sa; 3 Department of Pharmaceutics and Industrial Pharmacy, College of Pharmacy, Taif University, P.O. Box 11099, Taif 21944, Saudi Arabia; mrahman@tu.edu.sa (M.A.R.); mvarsi@tu.edu.sa (M.H.W.); 4 Department of Clinical Pharmacy, College of Pharmacy, Taif University, P.O. Box 11099, Taif 21944, Saudi Arabia; m.yusuf@tu.edu.sa; 5 Department of Pharmacognosy, College of Pharmacy, Prince Sattam Bin Abdulaziz University, P.O. Box 173, Al Kharj, Riyadh 11942, Saudi Arabia; prawez_pharma@yahoo.com

**Keywords:** lidocaine, nanogel, dermatokinetic, antioxidant, wound-healing

## Abstract

A wound refers to a cut or blow that may result in primary or secondary infection or even death, if untreated. In the current study, we have explored the wound-healing properties of lidocaine nanogel, owing to its antioxidant and neutrophilic modulatory potential. Initially, the pre-formulation study was performed and then using central composite design (CCD) fabrication and the characterization of lidocaine-loaded nanoemulsion was carried out. After the preparation of a nanogel of lidocaine-loaded nanoemulsion, it was evaluated on various parameters, such as pH, spreadability, extrudability, drug content, in vitro drug release, dermatokinetic study and in vivo skin safety. Based on the pre-formulation study, the maximum solubility of lidocaine was found in oleic acid (324.41 ± 4.19 mg/mL) and in Tween 20 (192.05 ± 8.25 mg/mL), selected as a suitable emulsifier. The refractive index of the optimized nanoemulsion was found to be 1.35 ± 0.04, the electrokinetic potential was recorded as −15.47 ± 0.95 mV. The pH, spreadability and extrudability of nanogel was found to be 6.87 ± 0.51, 73.32 ± 4.59 gm.cm/sec and 107.41 ± 6.42 gm/cm^2^, respectively. The percentage of the cumulative drug content and drug release from nanogel was found to be 99.94 ± 1.70% and 93.00 ± 4.67%, respectively. Moreover, dermatokinetic study showed significantly (*p <* 0.0005) improved drug deposition and the in vivo skin safety study showed no sign of dermal erythematous lesion or any visible damage. Stability studies also testified the secureness of nanogel after storage in a prescribed environmental condition. Thus, this study provides substantial evidence for healing wounds effectively and the further evaluation of the in vivo model. The patent related to this work was published in the Indian *Official Journal of the Patent Office* (Issue number: 20/2022).

## 1. Introduction

A wound refers to an injury in the skin caused by a cut, breakdown or blow that results in a laceration, loss of connective tissue, inflammation and bleeding [1]. It is reported to be one of the primary reasons for the loss of skin defence functions. According to WHO, the postoperative wound is a major contributor to mortality [2]. Wounds are considered a major confounding factor for patients’ poor quality of life and lay down an enormous burden on socio-economic balance. A report using retrospective market research states that the cost of management and treatment ranges from USD 28.1 billion to USD 96.8 billion worldwide, showing an exponential increment in the cost burden [3]. Wounds are generally classified as acute and chronic. Acute wounds are the result of chemical exposure, a cut, mechanical injury or burn. Whereas chronic wounds are often the result of a postoperative procedure or seen in coexisting disease conditions, such as diabetes. Additionally, wounds are also classified according to their depth. They are (a) superficial, (b) partial-thickness and (c) full-thickness wounds [4]. Pain, inflammation, bacterial infection and immobility are the primary consequences of a wound, and if not treated medically it will often precipitate hematoma, osteomyelitis, peri-wound edema and wound dehiscence and become fatal [5]. Wound healing is a complex and multifactorial process that requires the role of various cellular and molecular machinery, such as fibrin, the release of inflammatory mediators and the production of extracellular matrix (ECM), growth factors and collagens [6]. Currently, antibacterial drugs, such as cephalosporin or polymyxin B, and surgical dressing, hydrogels, sponges, etc., are routinely used to avoid the infection and to promote fast healing [6]. However, the use of orally administered drugs results in adverse effects due to multiple-dose regimens, fast hepatic metabolism and side effects [7]. Local anaesthetic, such as lidocaine, is one of the major add-on components along with antibacterial drugs which relieve pain. Much published evidence has reported lidocaine, a potent antioxidant effect, in in vivo studies [8,9]. As it is well established, during the pathology of wound several factors, such as thrombin, cytokines, TGF-β, serotonin, etc., play a pivotal role. Neutrophilic and monocytic infiltration is also reported to be involved in wound aetiology. Neutrophils are initial cells that invade the wound, increase vascular permeability because of the inflammation and formation of prostaglandins. Considering the impact of local anaesthetic in wound healing, the mitigation of neutrophilic infiltration, the modulation of surrounding pH, the direct effect on eicosanoids, the formation of fibroblast and antioxidants effects are noteworthy [10]. Moreover, when local anaesthetics are used, apart from relief from pain, anti-inflammatory effects have also been observed, which manifest into the wound healing [11,12]. Studies have also shown that local anaesthetics mitigate the adhesion of leukocytes to the wall of blood vessels, stimulate the release of prostacyclin and inhibit the enzymatic activity of phospholipase A2, leading to timely wound healing [13]. Vasseur et al., in 1984, explored the wound-healing potential of lidocaine and bupivacaine against abdominal wound-healing in rabbits. The outcome of the study showed that none of the drugs improved the healing of midline abdominal incisions [14]. In 2014, Abrão et at. also studied bupivacaine and ropivacaine for a possible wound-healing effect but no significant outcome was found [15].

The clinical relevance of local anaesthetic in wound-healing was reported by Hanci et al. in 2012 where the use of lidocaine and bupivacaine showed a reduction in the production of collagens and also reduced wound-breaking strength [16]. Excess neutrophilic infiltration and migration are the major factors that aggravate inflammation and result in delayed wound-healing [17]. Interestingly, apart from being a potent antioxidant drug, lidocaine inhibits, leukocytes’ excess infiltration and migration, and exhibits bactericidal action. Hence, lidocaine could be a potent repurposed drug for managing and treating wounds [18,19]. However, when 0.5/1% of lidocaine was studied for its wound-healing potential, the protective and positive effects on local inflammatory and proteolytic factors were observed through histopathological changes [20,21]. Hence, for the topical delivery of lidocaine nanoemulsion, nanogel has been developed to cross the skin barrier. This approach was hypothesized to enhance its permeability and bioavailability across the skin. For this purpose, a central composite design (CCD) was developed using experimental design software 11.0.5.0 (Stat-Ease Inc. Minneapolis, MN, USA), to provide the best possible nanoemulsion formula.

The current study was designed to develop lidocaine-loaded optimized nanoemulsion using the principle of CCD and was converted into a nanoemulsion-based nanogel. Prepared nanogel will offer several advantages over the conventional lidocaine gel in wound-healing, such as enhanced permeation across paracellular and transcellular spaces, increased bioavailability, increased C_max,_ T_max_ (h), AUC_0–12 h_ (mg/cm^2^h) and K_e_ (h^−1^) in the dermis and epidermis, respectively. These enhanced pharmacokinetic attributes will result in the increased drug concentration of the nanogel in the epidermis and dermis, respectively, as compared to the conventional formulation. The cumulative effect would lead to timely and improved wound-healing.

Hence, in the present study, after the evaluation of the nanogel in terms of spreadability, extrudability, drug content, etc., release patterns and ex vivo dermatokinetic studies were carried out in order to record the drug deposition across the skin layers. Additionally, a skin safety study was performed to ensure compatibility with the skin.

## 2. Results and Discussion

### 2.1. Selection of Suitable Excipients

**Figure 1:**
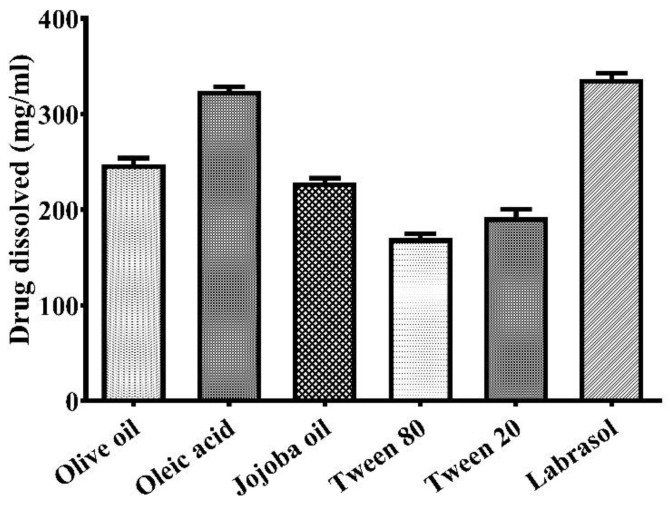
Solubility of lidocaine in various excipients. Data submitted as mean ± SD (*n* = 3).

A suitable oil and emulsifier was selected for the preparation of the nanoemulsion, based on better solubility, clarity and without the danger of precipitation. Various oils, such as olive oil, oleic acid, jojoba oil and emulsifiers (e.g., Tween 80, Tween 20 and labrasol), were analysed for the maximum solubility of lidocaine (Figure 1). From the results of the study, the maximum solubility of lidocaine was found in oleic acid (324.41 ± 4.19 mg/mL). Therefore, oleic acid was selected as the oil phase. Moreover, due to the maximum solubility of the drug in oleic acid, it prevents drug precipitation. However, Tween 20 (192.05 ± 8.25 mg/mL) was selected as a suitable emulsifier based on its better compatibility. Nevertheless, the maximum solubility of lidocaine was found in labrasol (336.82 ± 5.91 mg/mL). Slight turbidity was recorded in the solution of labrasol after 24 h of storage. In addition, the solubility of the drug in Tween 80 was 170.08 ± 4.84 mg/mL, which was comparatively lesser than in Tween 20 and labrasol. Thus, oleic acid was selected as the oil phase and Tween 20 as the emulsifier. The previous reports also supported these results [22].

### 2.2. Optimization and Statistical Analysis of Variables

**Figure 2:**
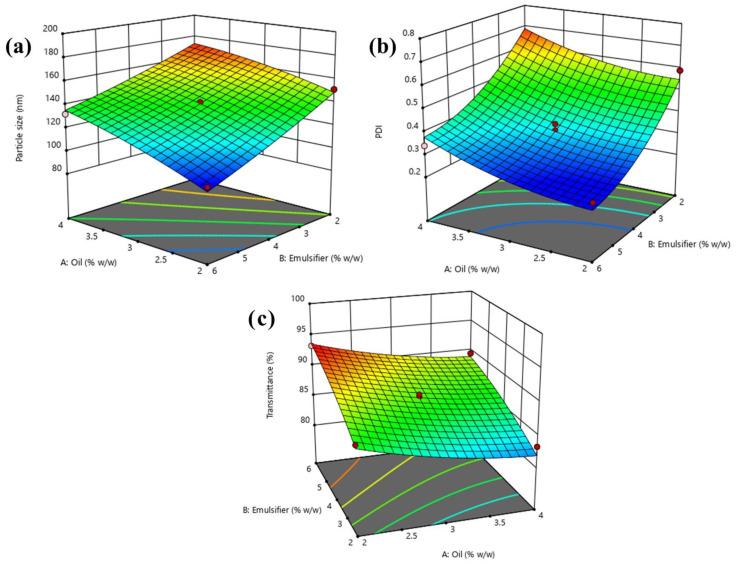
Three-dimensional response surface plots showing the simultaneous impact of independent variables on response parameters: (**a**) particle size, (**b**) PDI and (**c**) percent transmittance of lidocaine-loaded nanoemulsion within CCD experimental design.

A CCD-based statistical design was employed for the fabrication and optimization of lidocaine-loaded nanoemulsion by applying experimental design software **(**Table 1). All the possible effects of independent variables were evaluated by fitting the data on different experimental design models, where the quadratic model appeared as the best-fitted model (*p* < 0.05). The results obtained through the regression study of dependent variables using a quadratic model is depicted in Table 2. Simultaneously, the three-dimensional graph (Figure 2) exhibits the effects of independent variables on dependent variables.

#### 2.2.1. Effect of Independent Variables on Particle Size of Nanoemulsion

The particle size of nanoemulsion acts as a vital factor for the permeation and release of drug at the diseased site. Therefore, it was beneficial to prepare a nanoemulsion of small particle size. The quadratic model (*p <* 0.0001) explained the resulted responses better than the other models after fitting with model F value 82.16, and a reasonable difference between the predicted R^2^ value (0.8870) and adjusted R^2^ value (0.9713) was found, which was less than 0.20 (Table 3), thus indicating a suitable harmony between the two (Figure 2). The equation depicts the effect of independent variables on particle size.

Particle size = +139.80 + 15.67A − 22.19B + 3.25AB − 3.09A^2^ + 1.91B^2^ (1)

Equation (1) indicates the positive effect of oil, implying that the particle size of the nanoemulsion increases with the significant rise in the oil concentration. As the emulsifier showed a negative effect, this indicates the decrease in the particle size of the nanoemulsion due to the increase in the concentration of the emulsifier (Figure 2). Hence, the emulsifier reduced the interfacial tension between an oily and aqueous phase, thus being more miscible and resulting in smaller particle sizes.

#### 2.2.2. Effect of Independent Variables on PDI of Nanoemulsion

PDI acts as a crucial parameter in stabilising nanoemulsion. In nanoemulsions, the minimum PDI value is less than 0.30 and indicates a monodispersed nanosystem that is required for stability [23]. Therefore, it was beneficial to prepare a nanoemulsion with a minimum PDI. By applying analysis, the obtained data was practically analysed and fitted into the quadratic model. The outcomes declared that the quadratic model was the best fit model and ANOVA analysis declared its significance in terms of *p*-value (*p <* 0.0005) and F-value model (34.65). Furthermore, a reasonable difference between the predicted R^2^ value (0.7558), the adjusted R^2^ value (0.9334) was less than 0.20 and the following quadratic equation was achieved:

PDI = +0.3560 + 0.0748A − 0.1603B + 0.0050AB + 0.0351A^2^ + 0.0676B (2)

Equation (2) indicates the positive effect of oil and negative effect of the emulsifier on the PDI of nanoemulsion. The decreased PDI value was obtained with increased emulsifier concentration and the reduced concentration of oil (Figure 2). In this event, the maximum quantity of oil maximized the possibility of droplet aggregation, and on the other hand, increased emulsifier concentration decreased the droplets aggregation. Thus, the ratio of oil and emulsifier, which constitutes 2.17:5.35, showed a better PDI for nanoemulsion.

#### 2.2.3. Effect of Independent Variables on Percent Transmittance

The percent of transmittance is dependent on the particle size and is considered as an analytical tool to determine the clarity of a nanoemulsion. The maximum transmittance indicates the minimum particle size of the nanoemulsion. Therefore, obtained data were analysed by ANOVA and a statistically significant (*p <* 0.005) quadratic model was achieved with an F-value of 14.9. Furthermore, a reasonable difference between the predicted R^2^ value (0.7599), the adjusted R^2^ value (0.9324), was less than 0.20, and a further quadratic equation was obtained.

Transmittance = +87.66 − 2.30A + 3.52B − 0.0175AB + 0.8056A^2^ − 0.7594B^2^ (3)

Equation (3) clearly demonstrates the effect of emulsifier concentration. The maximum emulsifier concentration was beneficial for higher transmittance (Figure 2). Therefore, it is apparent that the maximum amount of emulsifier reduced the particle size, leading to the formation of a monodispersed nanosystem. Additionally, oil concentration exhibited a positive impact on the percent of transmittance. This signifies that the percent of transmittance decreases with the increment of oil concentration due to the formation of oil coalescence [24].

#### 2.2.4. Selection of Optimized Formulation

**Figure 3:**
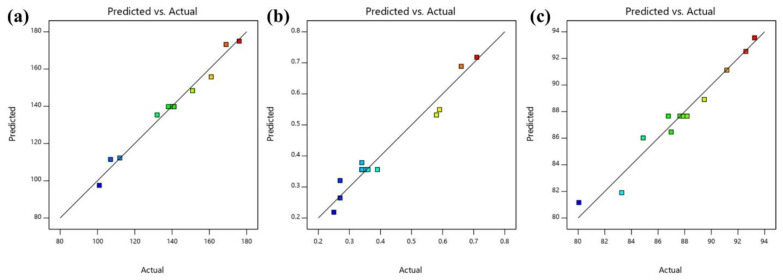
Linear correlation graph between predicted and actual outcomes (**a**) particle size, (**b**) PDI and (**c**) percent transmittance.

The optimized lidocaine-loaded nanoemulsion was selected from the criteria of achieved minimum particle size, PDI and maximum percent transmittance using the point prediction method of experimental design software. After analysing the different response variables and ambient evaluation, it was found that the nanoemulsion comprises of 1.59% *w*/*w* oil and 4% *w*/*w* emulsifier, fulfilling the need for an optimum formulation (Run no. 11). As a result, the optimized lidocaine-loaded nanoemulsion demonstrated a particle size of 107 nm with the PDI of 0.27. The percent of transmittance of the optimized nanoemulsion was found to be 92.59. Simultaneously, a quantitatively linear relationship was found between the responses of the experimental value and predicted value of all the dependent variables (Figure 3).

### 2.3. Evaluation of Optimized Nanoemulsion

However, nanoemulsions are generally treated as thermodynamically stable [25,26], but a thermodynamic stability study was performed to uncover the risk of precipitation, creaming, cracking and/or phase separation. As expected, the optimized lidocaine-loaded nanoemulsion easily passed all test parameters. The value of the refractive index acts as an indicator for the isotropic nature of nanoemulsion. The refractive index of the optimized nanoemulsion was found to be 1.35 ± 0.04, confirmed by the isotropicity of the formulation. The electrokinetic potential of the optimized nanoemulsion was recorded as −15.47 ± 0.95 mV due to the presence of a nonionic emulsifier in the formulation [27,28,29]. All the evaluation parameters demonstrates that the optimized nanoemulsion could be a rational drug delivery system for topical application in the form of semi-solid gel. Hence, a nanoemulsion-loaded nanogel of lidocaine was prepared and evaluated.

### 2.4. Evaluation of Nanogel

The nanogel prepared was evaluated for different parameters (results are depicted in Table 4). The gel formulations demonstrated an agreeable and homogeneous appearance without the presence of any gritty particles. The pH of nanogel and the conventional gel was 6.87 ± 0.51 and 6.93 ± 0.32, respectively. The pH obtained is considered to be safe in order to avoid any possibility of skin irritation upon application. Spreadability and extrudability are considered as the critical parameters for the uniform distribution and patient compliance of the gel formulation. The spreadability and extrudability of the nanogel obtained were 73.32 ± 4.59 gm.cm/s and 107.41 ± 6.42 gm/cm^2^, respectively, while the spreadability and extrudability of conventional gel were 70.42 ± 4.69 gm.cm/s and 114.81 ± 6.42 gm/cm^2^_,_ respectively. Simultaneously, the percent of drug content in the nanogel and conventional gel obtained were 99.94 ± 1.70 and 100.33 ± 2.08, respectively. Hence, prepared nanogel and conventional gel showed similar physical characteristic, which were required for further evaluation, such as in vitro release and a dermatokinetic and in vivo safety study.

#### 2.4.1. In Vitro Drug Release Study

**Figure 4:**
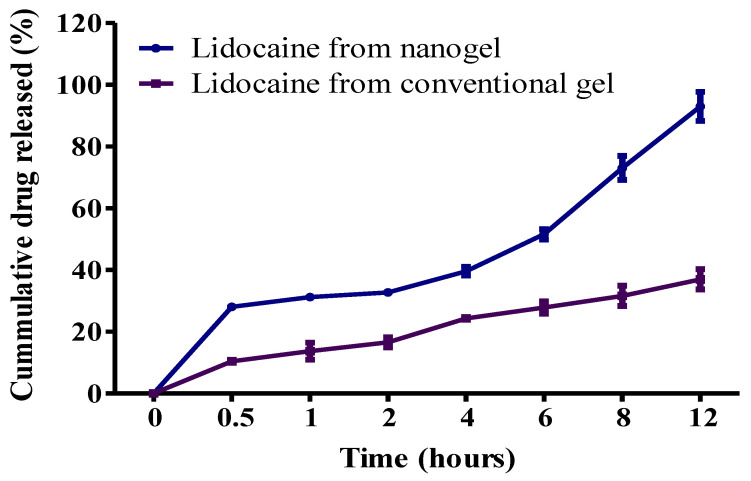
In vitro comparative release study of lidocaine-loaded nanogel and conventional gel.

The comparative in vitro release study was performed and quantified followed by the cumulative drug release. The cumulative drug release from nanogel and conventional gel was found to be 93.00 ± 4.67% and 36.97 ± 3.28%, respectively, after 12 h of in vitro release study (Figure 4). Results clearly indicated a significantly (*p <* 0.05) continuous sustained release from the optimized nanogel compared to the conventional gel. Nanogel showed a sustained drug release with initial burst release. The initial burst discharge occurred due to the presence of nano droplets on the surface of the nanoemulsion, while further sustained release was found as originating from the deliverance of drug from the oily core of the nanoemulsion, on account of the obstruction of the dialysis membrane [30]. Conversely, the restricted release of the drug from the conventional gel was due to the size of the drug molecules, membrane and aqueous medium, which collectively intercepted drug release. Thus, based on the outcome of the drug release study, nanogel exhibited superiority in the sustained release of drug with in vitro initial burst release, needed for dermal drug delivery in wound-healing.

#### 2.4.2. Dermatokinetic Study

**Figure 5:**
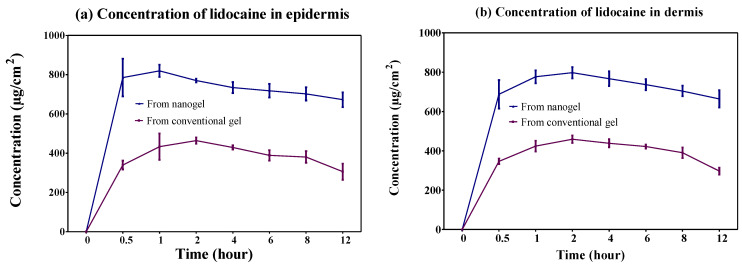
Dermatokinetic profile (mean ± SD), demonstrating lidocaine concentration in (**a**) epidermis and (**b**) dermis.

The drug availability in the epidermis and dermis layer of animal skin from the optimized nanogel and conventional gel is shown in Figure 5. The outcomes of the dermatokinetic study demonstrated significantly better results (*p <* 0.0005) in the epidermis and dermis layer of the skin for the nanogel compared to the conventional gel. The reason behind this favourable result of improved permeation was the nanosized globules and occlusive effect of the nanogel over the application area. The results of different dermatokinetic parameters are depicted in Table 5. An increased amount of the drug was detected in the skin layers from the optimized nanogel, which would be effective for wound-healing.

#### 2.4.3. In Vivo Skin Safety Study

In vivo skin safety studies of the nanogel and the conventional gel were performed in terms of skin irritation at the application site. This study exhibited zero scores for both formulations after two weeks. Neither the formation of edema nor erythema was detected, which indicated that the optimized nanogel is a safer drug delivery system for topical application.

#### 2.4.4. Stability Studies

**Figure 6:**
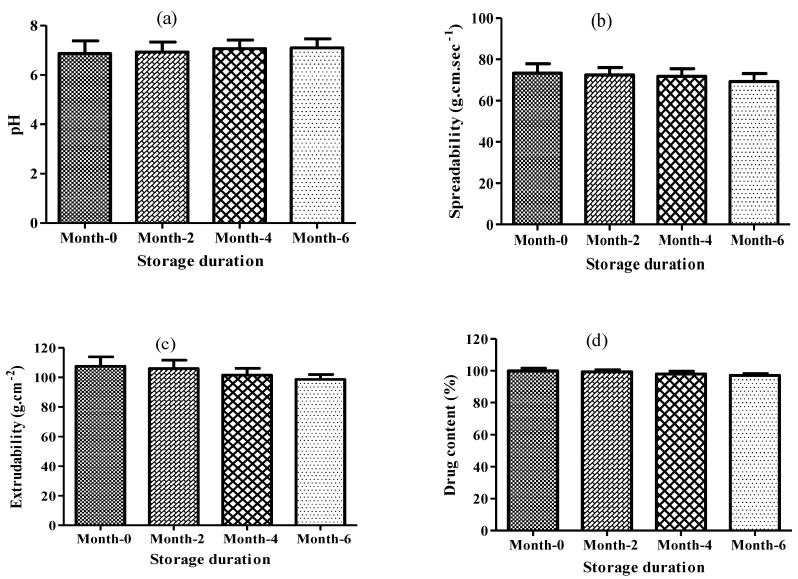
Stability studies (**a**) pH, (**b**) spreadability, (**c**) extrudability and (**d**) drug content.

In order to evaluate the stability behaviour of nanogel, a stability study was performed. No significant changes were observed in macroscopic, physical and content uniformity in the nanogel during storage (Figure 6). So, the result declared the exquisite stability of nanogel within the prescribed time period.

## 3. Conclusions

The present study was designed to explicate the optimization and fabrication of lidocaine nanogel for wound-healing. Initially, lidocaine-loaded nanoemulsion was optimized using the CCD design of the experiment through the characterization of different parameters, such as particle size, PDI and transmittance. Furthermore, optimized lidocaine nanoemulsion was converted into a gel using carbopol-940 as a gelling agent. The lidocaine-based nanogel showed improved thermodynamic stability, refractive index, electrokinetic potential, pH, spreadability, extrudability and drug content. The in vitro release study showed favourable results for the nanogel as compared to the conventional gel. Additionally, the nanogel exhibited a comparatively better dermatokinetic profile than the conventional gel. Additionally, the in vivo safety study showed no sign of toxicity after the topical application and was found to be safe for topical use. However, the exploration of a detailed in vivo study and the mechanism involved in wound-healing activity was suggested for bringing this formulation from bench to bedside.

## 4. Materials and Methods

### 4.1. Materials

Lidocaine was collected as a gift sample from Sun Pharmaceutical Industries Ltd., Gurugram, India. Labrasol was received as gift sample from Gattefosse. Olive and Jojoba oil was purchased from the local market, Delhi, India. Tween 20 and Tween 80 were purchased from Sigma-Aldrich. All other chemicals and reagents used in this study were of analytical grades.

### 4.2. Solubility Studies for the Selection of Suitable Excipients

The solubility of lidocaine in various excipients (oils and surfactants) were determined based on previously reported procedures [31]. Briefly, lidocaine was added in excess amounts to 1 mL of the selected oils/surfactants in 2 mL capacity stoppered vials and vortexed for 30 min (Nirmal International, Delhi, India). Then, the vials were kept at 25 ± 1 °C in a mechanical shaker incubator (Nirmal International, Delhi, India) for 72 h to reach equilibrium. After 72 h, the samples were removed from the shaker and centrifuged at 3000 rpm for 20 min (REMI International, Mumbai, India). Finally, the maximum solubility of lidocaine was determined from the collected supernatant spectrophotometrically at 260 nm [15].

### 4.3. Fabrication and Optimization of Lidocaine Nanoemulsion

The optimization of lidocaine-loaded nanoemulsion was undertaken using CCD, as shown in Table 1. The concentration of oleic acid and Tween 20 were chosen as independent variables and particle size, PDI and transmittance were taken as the dependent variables. The experimental design was generated and evaluated using experimental design software 11.0.5.0 (Stat-Ease Inc. Minneapolis, MN, USA) [24]. The lidocaine was dissolved in oleic acid, then the aqueous phase of the emulsifier was introduced and vortexed for 15 min for instant mixing. Furthermore, the specified volume of the double-distilled water was poured into the mixture drop-wise with continuous vortexing [26]. Finally, to obtain the lidocaine-loaded nanoemulsions, the prepared coarse nanoemulsion was sonicated using a UP50H^®^ ultrasonic processor (Hielscher-Ultrasonics, Teltow, Germany) with 40% amplitude for 60 s. The prepared nanoemulsion was characterized for selected parameters, such as particle size, PDI and percent transmittance to select the optimized nanoemulsion [32].

### 4.4. Characterization of Optimized Nanoemulsion

#### 4.4.1. Determination of particle Size, PDI and Percent Transmittance

The particle size and PDI of the nanoemulsion was determined by the dynamic light scattering method (Malvern Instruments Ltd, Worcestershire, UK). Nanoformulations were diluted 50 times using double-distilled water before analysis. The scattering was fixed at 90° and the temperature of the analytical samples were maintained at 25 ± 2 °C [1]. Additionally, the percent of transmittance was analysed using UV–Vis double beam spectrophotometer without the dilution of the nanoemulsion. Double-distilled water was employed as a blank [30].

#### 4.4.2. Determination of Thermodynamic Stability

The thermodynamic stability of the optimized nanoemulsion was studied through centrifugation, heating–cooling and freeze–thaw cycles (six cycles each). For the heating–cooling cycle, the sample was stored for 24 h between 45 °C and 25 ± 2 °C, followed by centrifugation at 5000 rpm for 30 min. Whereas in the freeze–thaw cycle, the sample was stored for 24 h between −20 °C and 25 ± 2 °C [25].

#### 4.4.3. Determination of Refractive Index

The refractive index of optimized nanoemulsion was recorded by an Abbe type refractometer. The recordings were carried out in triplicate [28].

#### 4.4.4. Determination of Zeta Potential

The optimized nanoemulsion (0.5 mL) was diluted 100 times using double-distilled water and the zeta potential was analysed in triplicate at 25 ± 1 °C using a Nano Zetasizer [28].

### 4.5. Development of Drug-Loaded Nanogel

The lidocaine-loaded nanoemulsion-based nanogel was prepared by the dispersion of 1% (*w*/*w*) Carbopol-940 into optimized nanoemulsion with continuous stirring. The prepared dispersion was kept for 24 h eliminating the air present inside and giving the gelling agent sufficient time for cross-linking to produce a clear gel. Then, a few drops of triethanolamine were added to adjust the pH of the nanogel formed for topical application. Moreover, in the preparation of the lidocaine conventional gel, the drug was initially dispersed in a small quantity of water, then spread on the swelled Carbopol-940 (1.25% *w*/*w*) for an even distribution of lidocaine in the gel medium. Further, some drops of triethanolamine were added to adjust the pH of the conventional gel form for topical application. The prepared nanogel and conventional gel were evaluated for pH measurement, drug content, drug release and skin permeation [1,33,34].

### 4.6. Evaluation of Nanogel

#### 4.6.1. Morphological and pH Determination

The optimized nanogel and conventional gel formulation was analysed for colour, appearance, washability and homogeneity by visual inspection. For the determination of grittiness, a small quantity of the gel formulation was pressed between the thumb and index finger. The homogenous gel formulation exhibited slippery behaviour with the privation of gritty particles. Simultaneously, pH was assessed using a digital pH meter (Mettler Toledo, Japan) at 25 ± 1 °C by dispersing 1 g of gel in 100 mL of double-distilled water and kept for 2 h for equilibrium attention. Furthermore, a pH electrode was dipped into the formulations and pH was recorded [35].

#### 4.6.2. Determination of Spreadability and Extrudability

The spreadability and extrudability of the gel formulation was analysed based on the reported method. Briefly, 1 g of gel was utilized for spreadability and 10 g for extrudability [36,37]. The analysis was carried out in triplicate. In order to carry out the spreadability efficiency study of the gel formulations, the weighed quantity of the sample was applied to one glass slide, and another glass slide was placed on top of the gel in such a way as to sandwich the sample between the two glass slides. The samples were then pressed between the upper and bottom glass slides using 100 g of weight, creating a consistent thin coating, and a portion of the excess sample was removed. Only the upper glass slide was removed effortlessly after being tied with a 20 g weight, as the other slide was securely fastened to the platform with minimal disruption. Spreadability was calculated from the length of time taken to slide the upper glass 7.50 cm over the thin sheet on the lower glass slide. Whereas to determine the extrudability of prepared gel formulations, the amount of gel extruding from collapsible tubes was evaluated. A weighed amount of each of the two types of gel formulation, i.e., nanogel and conventional gel, were placed in collapsible tubes. After that, the extrudability (g cm^−2^) was calculated based on the weight (g) necessary to extrude a 1 cm ribbon of the formulations from the collapsible tubes.

#### 4.6.3. Determination of Drug Content

Drug content was analysed by transferring the required quantity of methanol to dissolve the nanogel, followed by sonication for 20 min using a bath sonicator. The sample was filtered through a 0.45-micron membrane and quantified spectrophotometrically at 260 nm. The quantified amount of the drug present was given in percent.

#### 4.6.4. In Vitro Drug Release Study

The dialysis membrane of the molecular weight cut off between 12,000 to 14,000 Da was employed for the drug release study [38]. The dialysis membrane was fixed in between the acceptor and donor chamber of Franz diffusion cells, which had a cross-sectional area of 3.142 cm^2^. Amounts of 1 g of nanogel and conventional gel were applied to the donor chamber. In contrast, the receiver chamber was filled with a 30 mL solution of phosphate buffer saline (pH 7.4) and 30% (*v*/*v*) of methanol. Methanol was used to maintain the sink condition. The diffusion system was stirred continuously with the maintained temperature at 32 ± 1 °C. A 1 mL sample was replaced with an equal quantity of fresh diffusion solution at the predetermined time points (0.5, 1, 2, 4, 6, 8 and 12 h). The collected sample was filtered through a 0.45-micron membrane, and the released drug content was analysed at every time point spectrophotometrically at 260 nm [15].

#### 4.6.5. Dermatokinetic Study

The dermatokinetic study was performed as per the approval of the Research Ethics Committee (Approval no. 43-679), Taif University, Taif, Saudi Arabia. The study was performed using excised abdominal Wistar rat skin. Initially, the hair from the excised skin was removed with a surgical blade no. 22 and the skin was freed from any fatty materials. The skin was washed with normal saline and utilized for ex vivo dermatokinetic analysis. Finally, the excised skin was fixed in between the acceptor and donor chambers of the Franz diffusion cells, keeping the face of the stratum corneum towards the donor chamber. Amounts of 1 g of nanogel and conventional gel were applied to the donor and receiver chamber separately. The treated skin was removed at every prescribed time point, i.e., 0.0, 0.5, 1.0, 2.0, 4.0, 6.0, 8.0, and after 12 h, and washed with normal saline to remove the adhering formulation. The epidermis and dermis layers of skin were separated after soaking in warm water at 60 °C for 2–3 min and, finally, the separated layers of skin, i.e., epidermis and dermis, were macerated in methanol for 24 h to extract the drug. The sample was filtered through a 0.45-micron membrane and analysed for drug content spectrophotometrically at 260 nm. Further, the dermatokinetic parameters, such as the maximum drug concentration (C_max_), time to reach maximum drug concentration (T_max_), area under the curve (AUC_0–12 h_) and elimination rate constant (K_e_), were calculated using PK solver software, in which the deposited concentration of the drug at different selected time points was extrapolated against each other [1,29].

#### 4.6.6. In Vivo Skin Safety Study

The mice (4–6 weeks old; 20–25 g weight) were divided into two groups (3 animals in each group). The dorsal hair of animals was removed using a 0.1 mm animal hair clipper. The skins of the animals were shaved and wiped with saline wetted cotton 3–4 times. Each animal was kept in a separate cage with free access to food and water ad libitum. One group of animals was treated with topical nanogel and the other received the conventional gel. Both groups were treated with each formulation (1 g/day) for 2 weeks. Every day, the animals were inspected for the changes that appeared on the skin. At the end of the treatment, the residual formulation left on the skin was cleaned carefully with saline-soaked cotton. The skin surface was analysed keenly for the appearance of any edema or erythema with the help of a scoring scale. The grading scale constitutes the following values: erythema scale values (0, no erythema; 1, slight; 2, well defined; 3, moderate; 4, scar formation); edema scale values (0, no edema; 1, slight; 2, well defined; 3, moderate; 4, severe). Finally, the data obtained from the inspection were filtered [39,40].

#### 4.6.7. Stability Studies

The prepared lidocaine-loaded nanoemulsion-based nanogel was stored at 23 ± 2 °C for six months to determine the stability of the optimized nanogel [41]. Samples were collected routinely at 0, 2, 4 and 6 months and analysed in triplicate for visual investigation, pH, spreadability, extrudability and drug content [42].

### 4.7. Statistical Analysis

Data analysis was carried out using Graph pad Prism software v5.0 (Graph Pad Software San Diego, CA, USA). All experimental data were reported as mean ± standard deviation (SD). In addition, one-way ANOVA was used to analyse the results. The *p* value < 0.05 was considered statistically significant.

## 5. Patents

The authors have published a patent relevant to this work in the Indian *Official Journal of the Patent Office* (20/2022), (application number: 202211027483 A).

## Figures and Tables

**Table 1:** Selected independent variables for the optimization of lidocaine-loaded nanoemulsion using CCD.

Factors	Level Used	
**Independent variables**	**Low**	**High**
Oil (%*w*/*w*)	2	4
Emulsifier (%*w*/*w*)	2	6
**Dependent variables**	**Constraints**	
Particle size (nm)	Minimum	
PDI	Minimum	
Transmittance (%)	Maximum	

**Table 2:** Observed CCD experimental runs on lidocaine-loaded nanoemulsion with their actual and predicted experimental value of particle size, PDI and transmittance.

Run	Factor 1 A: Oil (% *w*/*w*)	Factor 2 B: Emulsifier (% *w*/*w*)	Response 1 Particle Size (nm)		Response 2 PDI		Response 3 Transmittance (%)	
			Actual	Predicted	Actual	Predicted	Actual	Predicted
1	3	6.82843	112	112.25	0.27	0.2646	91.16	91.12
2	3	1.17157	176	175.00	0.71	0.7179	80.05	81.16
3	3	4	141	139.80	0.35	0.3560	87.64	87.66
4	3	4	140	139.80	0.34	0.3560	87.82	87.66
5	4	2	169	173.23	0.66	0.6888	83.27	81.90
6	4.41421	4	161	155.79	0.58	0.5320	84.88	86.02
7	2	2	151	148.39	0.59	0.5492	86.98	86.47
8	2	6	101	97.52	0.25	0.2187	93.25	93.55
9	4	6	132	135.36	0.34	0.3783	89.47	88.91
10	3	4	138	139.80	0.34	0.3560	88.18	87.66
11	1.58579	4	107	111.46	0.27	0.3205	92.59	92.52
12	3	4	139	139.80	0.36	0.3560	87.89	87.66
13	3	4	141	139.80	0.39	0.3560	86.77	87.66

**Table 3:** Summary of regression study for responses, such as particle size, PDI and transmittance for CCD.

Response	Mean Square	Standard Deviation	R^2^	Adjusted R^2^	Predicted R^2^	Suggested Model
Particle size (nm)	1209.97	3.84	0.9832	0.9713	0.8870	Quadratic
PDI	0.0574	0.0407	0.9612	0.9334	0.7558	Quadratic

**Table 4:** Results of evaluation for nanogel and conventional gel.

Parameters	Nanogel	Conventional Gel
Colour	Creamy	White
Appearance	Translucent	Translucent
Washability	Good washability	Good washability
Homogeneity	Good	Good
pH	6.87 ± 0.51	6.93 ± 0.32
Spreadability (gm.cm/sec)	73.32 ± 4.59	70.42 ± 4.69
Extrudability (gm/cm^2^)	107.41 ± 6.42	114.81 ± 6.42
Drug content (Percent)	99.94 ± 1.70	100.33 ± 2.08

**Table 5:** Outcomes of various dermatokinetic parameters (mean ± SD) of lidocaine-loaded gel formulations.

Dermatokinetic Parameters	Nanogel		Conventional Gel	
	Epidermis	Dermis	Epidermis	Dermis
C_max_ (mg/cm^2^)	2.64 ± 0.02	2.53 ± 0.06	1.48 ± 0.02	1.46 ± 0.04
T_max_ (h)	0.83 ± 0.29	1.33 ± 0.58	1.33 ± 0.58	1.67 ± 0.58
AUC_0–12 h_ (mg/cm^2^h)	26.15 ± 0.92	26.39 ± 0.91	14.13 ± 0.49	14.40 ± 0.18
K_e_ (h^−1^)	0.016 ± 0.004	0.010 ± 0.001	0.02 ± 0.01	0.021 ± 0.004

## Data Availability

The data presented in this study are available on request from the corresponding author.
